# Peptide-Based Soft Hydrogels Modified with Gadolinium Complexes as MRI Contrast Agents

**DOI:** 10.3390/ph13020019

**Published:** 2020-01-21

**Authors:** Enrico Gallo, Carlo Diaferia, Enza Di Gregorio, Giancarlo Morelli, Eliana Gianolio, Antonella Accardo

**Affiliations:** 1 IRCCS SDN, Via E. Gianturco 113, 80143 Napoli, Italy; enricoglp@gmail.com; 2 Department of Pharmacy and Interuniversity Research Centre on Bioactive Peptides (CIRPeB), University of Naples “Federico II”, via Mezzocannone 16, 80134 Naples, Italy; carlo.diaferia@unina.it (C.D.); gmorelli@unina.it (G.M.); 3 Department of Molecular Biotechnology and Health Science, University of Turin, Via Nizza 52, 10125 Turin, Italy; enza.digregorio@unito.it (E.D.G.); eliana.gianolio@unito.it (E.G.)

**Keywords:** diagnostic agents, hydrogels, MRI contrast agents, peptide conjugates, in vitro cytotoxicity

## Abstract

Poly-aromatic peptide sequences are able to self-assemble into a variety of supramolecular aggregates such as fibers, hydrogels, and tree-like multi-branched nanostructures. Due to their biocompatible nature, these peptide nanostructures have been proposed for several applications in biology and nanomedicine (tissue engineering, drug delivery, bioimaging, and fabrication of biosensors). Here we report the synthesis, the structural characterization and the relaxometric behavior of two novel supramolecular diagnostic agents for magnetic resonance imaging (MRI) technique. These diagnostic agents are obtained for self-assembly of DTPA(Gd)-PEG8-(FY)3 or DOTA(Gd)-PEG8-(FY)3 peptide conjugates, in which the Gd-complexes are linked at the N-terminus of the PEG8-(FY)3 polymer peptide. This latter was previously found able to form self-supporting and stable soft hydrogels at a concentration of 1.0% wt. Analogously, also DTPA(Gd)-PEG8-(FY)3 and DOTA(Gd)-PEG8-(FY)3 exhibit the trend to gelificate at the same range of concentration. Moreover, the structural characterization points out that peptide (FY)3 moiety keeps its capability to arrange into β-sheet structures with an antiparallel orientation of the β-strands. The high relaxivity value of these nanostructures (~12 mM^−1^·s^−1^ at 20 MHz) and the very low in vitro cytotoxicity suggest their potential application as supramolecular diagnostic agents for MRI.

## 1. Introduction

Magnetic resonance imaging (MRI) is a diagnostic imaging technique currently used in clinic routine for a wide range of pathological conditions [1,2,3]. This technique is able to provide very well resolved images of the body, which are generated by signals related to the relaxation of water hydrogen nuclei excited by magnetic fields. The intrinsically high resolution of the images can be improved by providing suitable contrast agents (CAs). Thermodynamically stable and kinetically inert complexes of paramagnetic ions, such as gadolinium, are the most utilized MRI contrast agents; they generate a positive contrast (T_1_ CAs) in the images influencing the relaxation rate (R_1_ = 1/T_1_) of hydrogen nuclei of water molecules directly bounded to the paramagnetic ions or in their proximity [4,5]. The relaxation efficiency of a T_1_ CA is essentially affected by two parameters that are the molecular reorientation time (*τ*_R_) of the metal complex and the exchange lifetime (*τ*_m_) of the water molecules coordinated to the complex [6]. Low molecular weight contrast agents currently used in clinic are based on Gd-complexes stably coordinated by polydentate chelating agents with a linear structure such as diethylenetriaminepentaacetic acid (DTPA) or with a macrocyclic structure such as 1,4,7,10-tetraazacyclododecane-N,N,N,N-tetraacetic acid (DOTA). Indeed, in Europe, linear ligand based contrast agents have been recently withdrawn from the market due to possible toxicity risks connected to the Gadolinium retention in the body of patients who underwent multiple administrations of Gd-based contrast agents [7,8]. However, at the preclinical level, the study of DTPA-based contrast agents is still interesting because it allows to consider systems endowed with different structural/charge features with respect to macrocyclic complexes and draw important considerations on the structure–relaxivity relationship.

Commercial CAs show relaxivity values between 4 and 5 mM^−1^s^−1^ at 20 MHz and 310 K. In the past few years, many efforts have been made to improve CAs performance in terms of relaxivity values, biodistribution profile, and clearance time. One of the proposed strategies is the employment of macromolecular (polymers [9,10] and dendrimers [11,12]) or supramolecular (micelles [13,14,15], liposomes [16,17], nanogels [18,19], nanotubes [20,21], and fibers [22,23]) nanostructures for the delivery of Gd(III)-CAs. Due to their size and to their slow reorientation time, each gadolinium complex embedded in the nanostructure has a relaxivity value 2–5-fold higher in respect of the values associated to classical low molecular weight Gd-complexes. These supramolecular contrast agents can be obtained according to three different strategies: (i) Entrapping Gd(III) complexes within the internal compartments of the nanostructures, (ii) through covalent functionalization of inorganic nanostructures on their external surface or (iii) covalently linking the Gd(III) complexes to a hydrophobic portion able to prompt the self-assembly process. Polymeric, amphiphilic, and aromatic peptides are some of the potential molecular entities that could be used as aggregating systems to carry out these strategies [24]. For example, short aromatic peptides derivatized with Gd-DOTA or Gd-DTPA complexes self-assemble into water soluble fibrillary networks with a relaxivity value around 13–15 mM^−1^s^−1^ for each Gd-complex [22,23,25]. In this paper, we report on the synthesis, the structural characterization and relaxivity properties of self-assembled nanostructures obtained by an aromatic peptide eterosequence, (FY)3, derivatized with oxoethylene linkers (PEG8) and with Gd-DOTA or Gd-DTPA in order to investigate if the charge of the chelate affects hydrogel formation. This prospective was suggested by the evidence that PEG8-(FY)3 peptide is able to self-assemble in water into cross-*β* nanostructures, and above 1.0% wt, it forms a self-supporting and stable soft hydrogel with a G’ value of ~100 Pa [26]. Preliminary in vitro assays on cells indicated a very high cytocompatibility (cell survival = 93% after 24 h incubation). Now, we observed that the conjugation of a chelating agent in its free or complexed form do not change the capability of the aromatic peptide to generate hydrogels with a high in vitro biocompatibility. This study could represent an important step toward the obtainment of peptide-based gels containing the Gd(III)-complexes as injectable MRI contrast agent.

## 2. Results and Discussion

### 2.1. Synthesis and Structural Characterization

**Figure 1:**
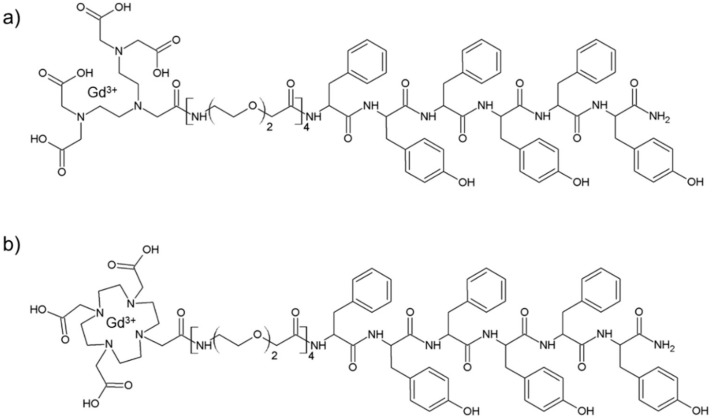
Schematic representation of DTPA(Gd)-PEG8-(FY)3 (**a**) and DOTA(Gd)-PEG8-(FY)3 (**b**) conjugates and structural characterization of self-assembled peptides in water solution.

**Figure 2:**
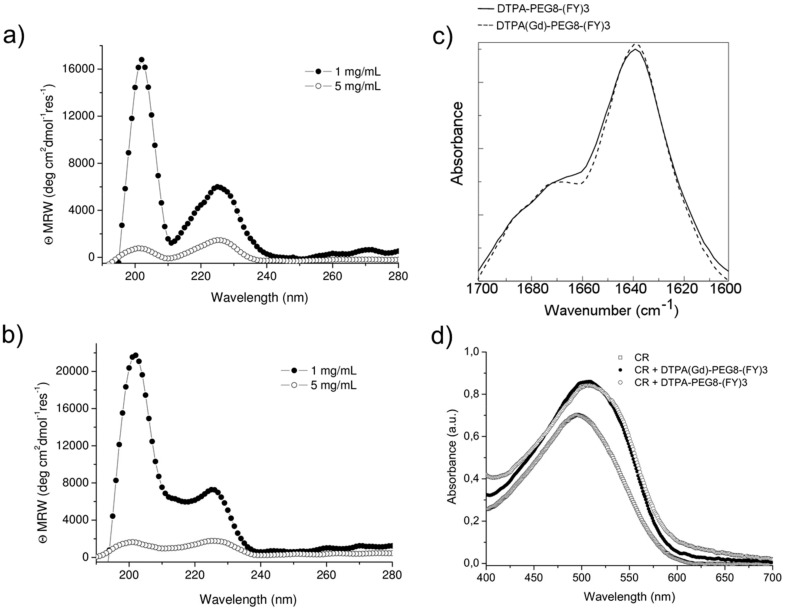
Structural characterization of peptide conjugates in solution. Far UV-CD spectra of DTPA-PEG8-(FY)3 (**a**) and DOTA(Gd)-PEG8-(FY)3 (**b**) at 1.0 and 5.0 mg/mL. (**c**) FTIR spectra in the amide I region and, (**d**) UV-Vis spectra of Congo red (CR) alone ore incubated with 1.5 mg/mL of the peptide conjugates as free based and as Gd-complexes.

Schematic representation of DTPA(Gd)-PEG8-(FY)3 and DOTA(Gd)-PEG8-(FY)3 peptide conjugates are reported in Figure 1. These monomers are composed by two functional regions: A chelating agent, to achieve the Gd(III) complexation, and a peptide-polymer [PEG8-(FY)3] to allow the process of self-aggregation. PEG8-(FY)3 moiety was synthesized according to the solid phase peptide synthesis (SPPS) with Fmoc/tBu strategy. Then, cyclic or branched chelating agents (DOTA or the DTPA) were coupled at the N-terminus of PEG-fragment directly in solid phase using their fully protected derivatives, DOTA(OtBu)_3_-OH and DTPA(OtBu)_4_-OH, respectively. At the end of the synthesis, the crude compounds were removed from the solid support in an acidic solution via trifluoroacetic acid (TFA) and precipitated with ice-cold ethyl ether. After purification step, peptides (purity > 97%) were characterized by ^1^HNMR and LC-MS (see Figure S1) in order to confirm their chemical identity. The complexation of gadolinium(III) ion to the peptide conjugates has been performed by adding equimolar amounts of GdCl_3_ to the aqueous solutions of the peptide derivatives (0.5 mg/mL) at pH 7.0 and 25 °C. As expected, both conjugates, before and after complexation, exhibit a good solubility in water (up to 12 mg/mL) and the formation of a translucent soft hydrogel begin to appear only above a concentration of 5.0 mg/mL. Below this concentration, which can be identified as the critical gelation concentration (CGC), the peptide begins to self-aggregate, generating a clear suspension. The hydrogel formation is consequence of a multiscale phenomenon in which small molecular entities self-organize into fibrillary nanostructures. Due to the mutual entangling and the physically crosslink, a tridimensional network, capable of immobilizing the aqueous solvent, takes place. A first evidence of the peptide self-assembly was highlighted by fluorescence measurements performed on solutions at different concentrations (see Figure S2). Spectra of DTPA(Gd)-PEG8-(FY)3 and DOTA(Gd)-PEG8-(FY)3 were recorded by exciting the peptide conjugates both at 257 and at 276 nm, that are the absorbance wavelengths for Phe and Tyr amino acids, respectively. As previously observed for PEG8-(FY)3, independently from the selected λ_ex_ (257 and 276 nm), the spectra show an emission peak at 305 nm only, due to the Tyr residue. Indeed, due to the RET (resonance energy transfer) phenomenon occurring between Phe and Tyr residues, we do not observe the emission peak of the Phe at 282 nm. From the inspection of spectra, we observe a quenching between 0.5 and 1.0 mg/mL, attributable to the stacking of the aromatic rings that occurs during the aggregation of the peptide moiety. This range of concentrations is in good agreement with that of the peptide polymer [PEG8-(FY)3], thus suggesting that the insertion of the chelating agent do not alter significantly the aggregation properties of the peptide [26]. In order to support this idea, structural characterization in aqueous solution of peptide conjugates in their self-assembled form was assessed by three complementary techniques: Circular dichroism (CD), Fourier transform infrared (FTIR), and Congo red (CR) spectroscopic assay. CD and FTIR spectroscopies allow obtaining accurate information for evaluating the secondary structure elements in peptide self-assembly [27,28]. Despite being characterized by different charge (DTPA-monoamide ligand introduces one negative charge while DOTA-amide ligand is neutral), the structural characterization performed on the DTPA and DOTA conjugates pointed out a very similar behavior for the two derivatives. This experimental result confirms that the effect of the complexes derivatization at the N-terminus of the peptide is negligible. According to these considerations, we decided to report only spectra related to the characterization of DTPA derivative and its Gd-complex. CD spectra of DTPA-PEG8-(FY)3 and DOTA-PEG8-(FY)3 solutions, and their corresponding Gd-complexes have been recorded between 280 and 190 nm at two different concentrations 1.0 and 5.0 mg/mL (see Figure 2a,b), below and above the CGC. All the spectra showed the same dichroic signature composed of two well distinct maxima around 226 and 202 nm and a minimum at 218 nm. This behavior was previously observed by us in CD spectra of PEG8-(FY)3 [26]. The two maxima are associable with n-π* and π-π* transitions of the peptide bond and of the peptide bond and/or the phenyl moieties, respectively. The minimum at 218 nm is associated with a β-sheet secondary structure arrangement, whereas the maximum at 202 nm is indicative of the stacking between the aromatic side-chains. The decrease of this latter peak as function of the peptide concentration (from 1.0 to 5.0 mg/mL) is due to the increase of the stacking occurring during the peptide self-aggregation. The β-sheet secondary structure of the peptide conjugates was also observed by FTIR spectroscopy. In Figure 2c the deconvolution in absorbance for the secondary structure prediction of the amide I spectral region (1600–1700 cm^−1^) is reported. FTIR spectra show two peaks around 1638 and 1680 cm^−1^, typical of β-sheet structures with antiparallel orientation of the β-strands. Due to the presence of residual traces of TFA sourced from the synthesis, there is a peak at 1672 cm^−1^ overlapped with the peak at 1680 cm^−1^. Beside the peaks in the amide I region, all the FTIR spectra show a broad band around 3460 cm^−1^ (see Figure S3). This band is ascribable to the stretch deformation occurring during the formation of H-bonding interactions between Tyr phenol groups and water molecules, peptide backbone, or other Tyr side chains present in the sample. Secondary structure of both peptide conjugates was further characterized by Congo Red (CR) assay. CR is an azoic dye able to reveal the presence in solution of β-sheet structures [29]. In Figure 2d, UV-Vis spectra show the spectral shift of the conventional CR band from 488 to 540 nm after its incubation with peptide conjugate solution at 1.5 mg/mL for 30 min. The change in the CR spectral behavior suggests the formation of supramolecular amyloid-like fibrils.

### 2.2. Scanning Electron Microscopy

**Figure 3:**
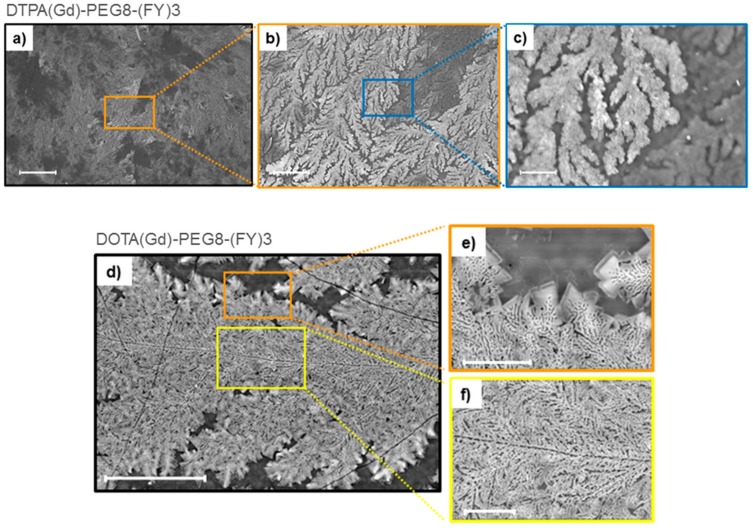
Selected SEM microphotos for DTPA(Gd)-PEG8-(FY)3 (**a**,**b**,**c**) and DOTA(Gd)-PEG8-(FY)3 (**d**,**e**,**f**) xerogels drop-casted from a solution at a concentration of 5 mg·mL*^−^*^1^: scale bars are 500 μm (**a**), 100 μm (**b**), 20 μm (**c**), 50 μm (**d**), 10 μm (**e**), and 10 μm (**f**), respectively.

Supramolecular organization of both peptide Gd-complexes DTPA(Gd)-PEG8-(FY)3 and DOTA(Gd)-PEG8-(FY)3 were also investigated by Scanning electron microscopy (SEM) technique. Microphotos of xerogels, drop-casted from 5.0 mg/mL solutions are reported in Figure 3. Both the samples show the formation of highly ordered tree-like multi-branch nanostructures with well-distinguished nucleation centers. These nanostructures appear completely different respect to the nanostructures previously observed for PEG8-(FY)3 in which are clearly visible continuous networks of fibers interconnected by physical cross-linking points [26]. This different morphology is originated by the steric hindrance of the metal complex.

### 2.3. Relaxivity Study

**Figure 4:**
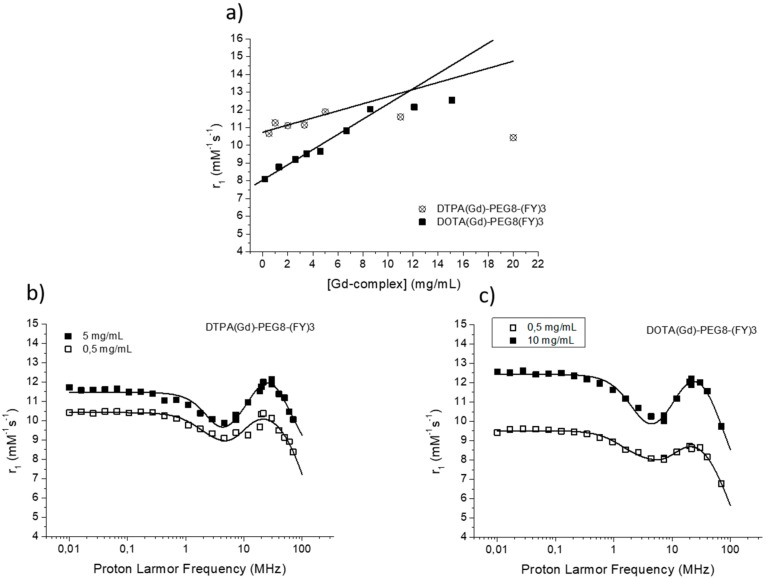
(**a**) Longitudinal proton relaxivity of DTPA(Gd)-PEG8-(FY)3 and DOTA(Gd)-PEG8-(FY)3 measured at 21.5 MHz (0.5 T), 298 K and pH = 7 as a function of the concentration of the Gd-complexes. (**b**) and (**c**) nuclear magnetic resonance dispersion (NMRD) profiles of aqueous solutions of DTPA(Gd)-PEG8-(FY)3 (0.5 and 5 mg/mL) and DOTA(Gd)-PEG8-(FY)3 (0.5 and 10 mg/mL) at 298 K and pH = 7. The data refer to 1 mM concentration of the paramagnetic complexes.

**Figure 5:**
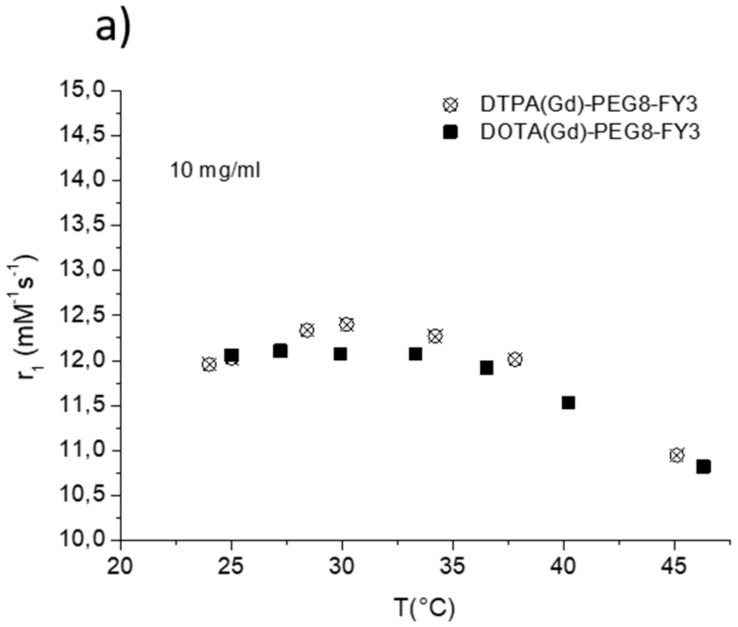

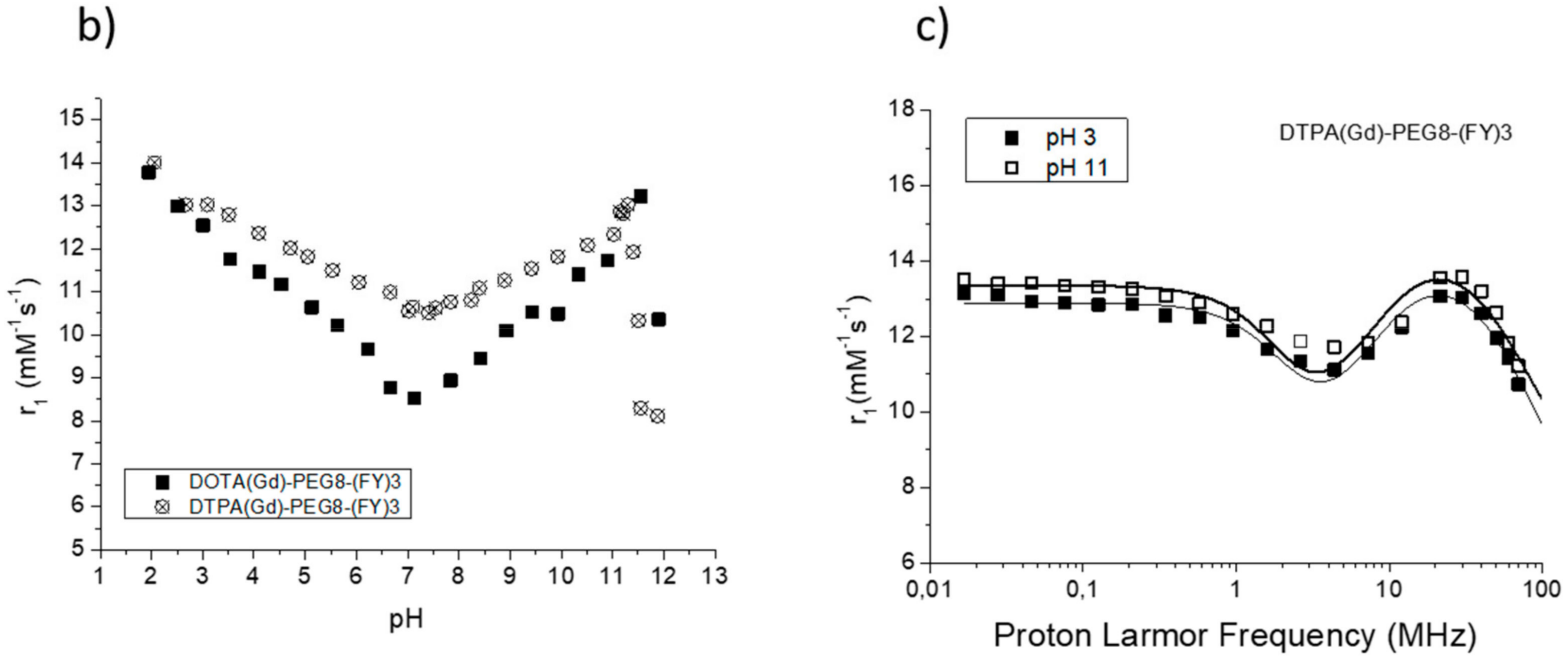
(**a**) Longitudinal proton relaxivity of DTPA(Gd)-PEG8-(FY)3 and DOTA(Gd)-PEG8-(FY)3 measured at 21.5 MHz (0.5 T) and pH = 7 as a function of temperature. (**b**) Longitudinal proton relaxivity of DTPA(Gd)-PEG8-(FY)3 and DOTA(Gd)-PEG8-(FY)3 measured at 21.5 MHz (0.5 T) and 298 K as a function of pH of the solution. (**c**) Nuclear magnetic resonance dispersion (NMRD) profiles of aqueous solutions of Gd-DTPA-PEG8-(FY)3 (0.5 mg/mL) at 298 K and pH = 3 and pH = 11. The data refer to 1 mM concentration of the paramagnetic complexes.

The efficacy of MR contrast agents is related to the ability to strongly enhance the water protons relaxation rate in tissues and aqueous solutions thanks to the magnetic dipolar interaction between unpaired electrons on the paramagnetic gadolinium ions and the water protons. This ability is usually defined as longitudinal “relaxivity” (r_1_) and is referred to the water proton relaxation rate of a solution containing one millimolar concentration of the Gd-complex. The relaxivity of a Gd-containing system depends on the complex interplay of structural, dynamic, and electronic parameters [6]. At the frequencies most commonly used in commercial tomographs (20–60 MHz), r_1_ is generally determined by the reorientational correlation time (τ_R_) of the chelate so that systems of higher size display higher relaxivity. Based on this property, it is possible to follow the formation of an aggregated system containing a Gd-complex through the measure of the water proton longitudinal relaxation rate of its aqueous solution. Figure 4a reports the relaxivities of DOTA(Gd)-PEG8-(FY)3 and DTPA(Gd)-PEG8-(FY)3 measured at 21.5 MHz (0.5 T), 298 K and neutral pH, as a function of the complex concentration. From the inspection of the graphic in Figure 4a, we can observe a progressive enhancement in relaxivity up to ca. 12.0 mM^−1^s^−1^ at 5.0 mg/mL for DTPA derivative and 12.1 mM^−1^s^−1^ at 10.0 mg/mL for DOTA one. This behavior is indicative of the self-aggregation degree of the two Gd-complexes. Above these concentrations (5.0 and 10.0 mg/mL for DTPA and DOTA, respectively), r_1_ assumes an almost stable value which corresponds to the relaxivity of systems in their gelyficated form. In the low concentration range, the relaxivity of DTPA(Gd)-PEG8(FY)3 is higher than that of DOTA(Gd)-PEG8-(FY)3 indicating increased tendency to aggregation despite it is characterized by one negative residual charge while DOTA(Gd)-PEG8-(FY)3 is neutral. The analysis of the magnetic field dependence of the relaxivity, obtained through the registration of the so called NMRD (nuclear magnetic resonance dispersion) profiles, allows the determination of the principal parameters characterizing the relaxivity of a Gd(III) chelate. The NMRD profiles of DTPA(Gd)-PEG8-(FY)3 and DOTA(Gd)-PEG8-(FY)3, measured at low and high concentration, are reported in Figure 4b,c. From a qualitative point of view, the shape of NMRD profiles gives information on the aggregation state of the system. Low molecular weight Gd-complexes, indeed, show NMRD profiles with a dispersion in the region 1–10 MHz, while a characteristic peak of relaxivity appears, in the region of proton Larmor frequencies 10–70 MHz, in the case of high molecular weight Gd-containing systems [6,30]. The shape of both the profiles relative to DTPA(Gd)-PEG8-(FY)3 (Figure 4b) is a clear indication that the Gd-complex is in aggregated form both at 0.5 mg/mL and 5 mg/mL, but the extent of aggregation increases as the concentration is increased. This result is in good agreement with fluorescence measurements carried out on the peptide conjugates at different concentrations. In the case of DOTA(Gd)-PEG8-(FY)3 (Figure 4c) the data obtained by measuring the NMRD profile at 0.5 mg/mL show a smoother peak in the 10–70 MHz range, supporting the minor tendency to aggregate. Data were fitted using the Solomon–Bloembergen–Morgan model [31,32], considering one water molecule in the inner coordination sphere (q = 1) and fixing the exchange lifetime (τ_M_) to a reliable value (700 ps) on the basis of those previously reported for mono-amido DOTA and DTPA small complexes [33,34,35]. Given that the aggregation takes place through the stacking of the peptide chains and the presence of a quite long chain connecting the peptide and Gd-complex prevents, in principle, crowding at the coordination cage, it was assumed that hydrogel formation does not affect water access to gadolinium and thus water exchange. This hypothesis was supported from the analysis of the temperature dependence of the proton relaxivities of DTPA(Gd)-PEG8-(FY)3 and DOTA(Gd)-PEG8-(FY)3 measured at 21.5 MHz, neutral pH and 10 mg/mL concentration (Figure 5a). For both systems, only a smooth increase in relaxivity is observed by increasing temperature, indicating that the water exchange has only a very limited effect on the quenching of the overall relaxivity.

The quantitative analysis of the NMRD profile of the aggregated form was carried out by using the Lipari-Szabo approach for the description of the rotational dynamics, which accounts for the presence of a certain degree of internal rotation superimposed on the overall tumbling motion [36,37]. These two types of motion, a relatively fast local rotation of the coordination cage about the linker to the peptide scaffold superimposed on the global reorientation of the system, are characterized by different correlation times: Local rotational correlation time (τ_R_^l^) and global rotational correlation time (τ_R_^g^), respectively. The degree of correlation between global and local rotations is given by the parameter S^2^, which can be comprised between 0 (completely independent motions) and 1 (entirely correlated motions). Both the shape of the profiles and the τ_R_ values determined from the fitting of the experimental results (Table 1) validate the hypothesis of the formation of aggregates. The values of τ_R_^g^ reported in Table 1 are indicative of smaller aggregates in the low concentration regime (τ_R_^g^ ≈ 1700–2000 ps) and more extended aggregates in the high concentration one (τ_R_^g^ ≈ 2700–3400 ps).

The high field relaxivity of the two complexes in the completely aggregated form are very close, being, at 21.5 MHz and neutral pH, 12.0 and 12.1 mM^−1^s^−1^ for DTPA(Gd)-PEG8-(FY)3 and DOTA(Gd)-PEG8-(FY)3, respectively. Exploring the trend of relaxivity of DTPA(Gd)-PEG8-(FY)3 and DOTA(Gd)-PEG8-(FY)3 (concentration = 0.5 mg/mL) as a function of the pH of the solution (Figure 5b, we observed an interesting and not expected behavior. For both the Gd-complexes, a minimum of relaxivity is observed at neutral pH while a steady increase in relaxivity occurs by decreasing (up to pH = 2) or increasing (up to pH = 11) the pH of the solution. Having excluded a limiting effect of a too slow exchange lifetime on the overall relaxivity at neutral pH, which could lead to evoke an acid- and basic-catalyzed proton exchange to justify this relaxivity enhancement, it was concluded that it can be ascribed to a pH promoted aggregation of the system even in the low concentration condition. This hypothesis is supported from the fitting of NMRD profiles of DTPA(Gd)-PEG8-(FY)3 (0.5 mg/mL) measured at pH 3 and 11 (Figure 5c. τ_R_^l^ and τ_R_^g^ reported in Table 1 are indeed very close to those found for the aggregated systems at neutral pH and higher concentration. 

### 2.4. Cytotoxicity Assays

**Figure 6:**
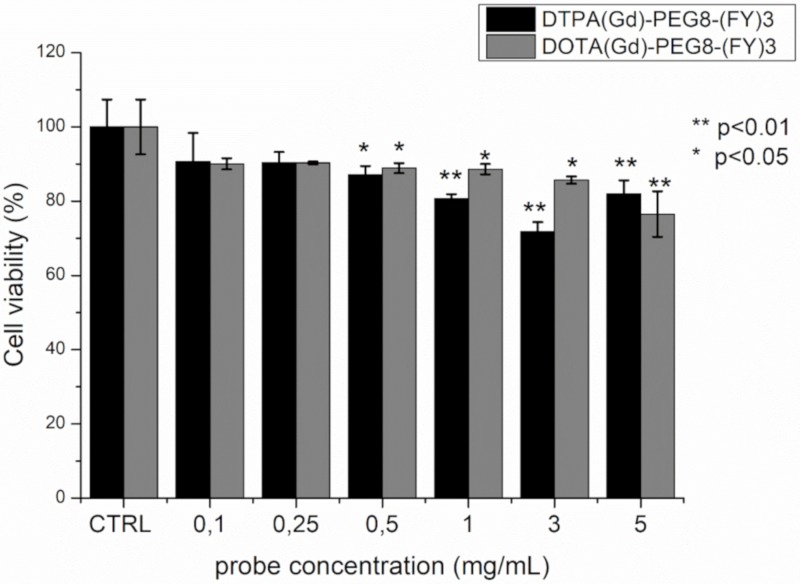
Cell viability percentage, evaluated by MTT assay, after 4 h incubation of TS/A mouse mammary adenocarcinoma with various concentrations of DOTA(Gd)-PEG8-(FY)3 and DTPA(Gd)-PEG8-(FY)3. Data are expressed as the mean ± SD (*n* = 3). Statistically different values with respect to control cells are indicated with * for *p* < 0.05 and ** for *p* < 0.01.

The cytotoxicity of DTPA(Gd)-PEG8-(FY)3 and DOTA(Gd)-PEG8-(FY)3 was investigated in the metastasizing TS/A mouse mammary adenocarcinoma cell line by incubating the cells with the probes in the concentration range 0.1–5 mg/mL for 4 h. Cell viability was tested by using the classical MTT assay. As can be observed in Figure 6, the presence of the two Gd-containing probes does not affect substantially cells viability. Even if a statistically significant reduction in viability is observed at concentrations approaching the formation of hydrogels, even at the highest concentration tested, the viability remains around 80%. This value is not considerably lower than those reported for clinically used Gd-based contrast agents, which, although generally considered safe, have cytotoxicity that is cell line dependent and case specific [38,39]. Indeed, a toxic effect of commercial Gd-containing contrast agents on mitochondrial respiratory function and cell viability has been recently demonstrated in cultured human neurons. Citotoxicity is dose dependent and increases as the kinetic stability of the contrast agent decreases [40].

Moreover, viability is higher with respect to other peptide-based hydrogels (such as Fmoc-FF). The good cytocompatibility profiles detected in this case can be attributed to the hydrogel preparation methodology. Indeed, contrarily to other peptide hydrogels, DTPA(Gd)-PEG8-(FY)3 and DOTA(Gd)-PEG8-(FY)3 gelification does not require the use of some organic solvents (like DMSO or MeOH) [41], preventing extensive cellular toxicity. 

## 3. Materials and Methods

Materials: Protected N^α^-Fmoc-amino acid derivatives, coupling reagents and Rink amide MBHA (4-methylbenzhydrylamine) resin were purchased from Calbiochem-Novabiochem (Laufelfingen, Switzerland). The monodisperse Fmoc-8-amino-3,6-dioxaoctanoic acid, [Fmoc-AdOO-OH,PEG2] was purchased from Neosystem (Strasbourg, France). DOTA(OtBu)_3_-OH and DTPA(OtBu)_4_-OH chelating agents were purchased from Chemateck (Dijon, France). All other chemicals were commercially available by Sigma-Aldrich (Milan, Italy) or Fluka (Bucks, The Switzerland) or LabScan (Stillorgan, Dublin, Ireland) and were used as received unless otherwise stated. All solutions were prepared by weight with doubly distilled water. Preparative RP-HPLCs were carried out on a LC8 Shimadzu HPLC system (Shimadzu Corporation, Kyoto, Japan) equipped with a UV lambda-Max Model 481 detector using Phenomenex (Torrance, CA, USA) C18 column. Elution solvents are H_2_O/0.1% TFA (A) and CH_3_CN/0.1% TFA (B), from 5% to 70% over 30 min at 20 mL/min flow rate. Purity and identity of the products were assessed by analytical LC-MS on a LC-MS Agilent Technologies 6230 ESITOF on a Phenomenex Jupiter 3 μ C18 (150 × 2.0 mm) column eluted with an H_2_O/0.1% TFA (A) and CH_3_CN/0.1% TFA (B) from 5% to 70% over 15 min at a flow rate of 0.2 mL·min^−1^.

### 3.1. Synthesis of Peptide Derivatives

DOTA-PEG8-(FY)3 and DTPA-PEG8-(FY)3 were synthesized as previously reported according to the standard solid-phase 9-fluorenylmethoxycarbonyl (Fmoc) protocols [42]. Briefly, (FY)3 peptide sequence was synthetized on Rink amide MBHA resin (substitution 0.65 mmol/g). Each amino acid was coupled twice for 45 min in N,N-dimethylformamide (DMF) in presence of the activating agents 1-hydroxybenzotriazole (HOBt), benzotriazol-1-yl-oxy-tris-pyrrolidino-phosphonium (PyBop) and diisopropylethylamine (DIPEA). After each coupling, Fmoc protecting group was removed from the N-terminus by treating twice the peptidyl-resin with DMF/Piperidine (70/30, v/v) for 10 min. Oxoethylene spacers, Fmoc-AdOO-OH, and chelating agents [DOTA(OtBu)_3_-OH or DTPA(OtBu)_4_-OH] were coupled as previously reported [23]. Peptide deprotections and cleavages from the solid support were achieved by stirring the peptidyl-resin in TFA (trifluoroacetic acid)/TIS (triisopropylsilane)/H_2_O (92.5/5.0/2.5 v/v/v) mixture at room temperature for 2 h. Crude peptide conjugates were precipitated with ice-cold ethyl ether, dissolved in H_2_O/CH_3_CN and lyophilized. Then, the crude products were purified by RP-HPLC chromatography and their purity and identity were confirmed by ESI mass spectrometry and ^1^HNMR spectroscopy.

### 3.2. DOTA-PEG8-(FY)3 Characterization

^1^H-NMR (CD_3_OD) (chemical shifts in *δ*, CH_3_OH as internal standard 3.55) = 7.48–7.35 (m, 15 C*H* aromatic Phe), 7.18–7.10 (m, 6 C*Hδ* aromatic Tyr), 6.88–6.85 (m, 6 C*H*ε aromatic Tyr),4.81–4.54 (m, 6H, C*H*α of Phe and Tyr), 3.83 (s, 16H, OC*H*_2_C*H*_2_O), 3.80 (t, 8H, RNH-CH_2_C*H*_2_O), 3.75–3.72 (m, 8H, OC*H*_2_COR), 3.70 (s, 6H, R2NC*H*_2_COOH), 3.63 (t, 8H, RNH-C*H*_2_CH_2_O), 3.45 (s, 16H, R2NC*H*_2_C*H*_2_NR2), 3.40–3.36 (m, 2H, R2NC*H*_2_CONH), 3.35–2.94 (m, 12H, C*H*_2_*β* of Phe and Tyr).

HPLC-retention time, R_t_ = 12.63 min; MS (ESI^+^): m/z 1915.0979 calcd. For C_94_H_127_N_15_O_28_: [M + H^+^] = 1915.8965; [M + 2H^+^]/2 = 958.4520.

### 3.3. DTPA-PEG8-(FY)3 Characterization

^1^H-NMR (CD_3_OD) (chemical shifts in δ, CH_3_OH as internal standard 3.55) = 7.48–7.35 (m, 15 CH aromatic Phe), 7.18–7.10 (m, 6 CHδ aromatic Tyr), 6.88–6.85 (m, 6 CHε aromatic Tyr), 4.81–4.54 (m, 6H, CHα of Phe and Tyr), 4.65–4.40 (dd, 2H, R2NCH_2_CONHR), 3.87 (s, 8H, R2NCH_2_COOH), 3.83 (s, 16H, OCH_2_CH_2_O), 3.80 (t, 8H, RNH-CH_2_CH_2_O), 3.75–3.72 (m, 8H, OCH_2_COR), 3.63 (t, 8H, RNH-CH_2_CH_2_O), 3.53–3.50 (m, 4H, R2NCH_2_CH_2_NR2), 3.42–3.31 (m, 4H, R2NCH_2_CH_2_NR2), 3.35–2.94 (m, 12H, CH_2_β of Phe and Tyr).

HPLC-retention time, R_t_ = 12.82 min; MS (ESI^+^): m/z 1904.0289 calcd. For C_92_H_122_N_14_O_30_: [M + H^+^] = 1904.0520; [M + 2H^+^]/2 = 953.0571.

### 3.4. Preparation of Gadolinium Complexes

Gadolinium complexes were prepared through the addition of GdCl_3_ to the aqueous solutions of the DOTA-functionalized ligands in a molar ratio 1:1 at room temperature and neutral pH. The absence of any residual free Gd(III) ions was checked by the orange xylenol UV method, the eventual residual was complexed by addition of the corresponding amount of ligand.

### 3.5. Preparation of Peptide Conjugate Solutions and Hydrogels

Peptide conjugate solutions were prepared by dissolving the lyophilized powder in double distilled water. The concentration of the final solution was estimated by absorbance on UV-vis Thermo Fisher Scientific Inc (Wilmington, DE, USA) Nanodrop 2000c spectrophotometer equipped with a 1.0 cm quartz cuvette (Hellma) using a molar absorptivity (ε_276_) of 4230 M*^−^*^1^cm*^−^*^1^.

### 3.6. Fluorescence Studies

Fluorescence spectra were recorded at room temperature on a Jasco Model FP-750 spectrofluorophotometer in a 1.0 cm path length quartz cell, using equal excitation and emission bandwidths (5 nm) with a recording speed of 125 nm min*^−^*^1^ and automatic selection of the time constant. Fluorescence emission of both derivatives was studied for peptide water solutions at several concentrations (0.1, 0.5, 1.0, and 2.5 mg/mL) exciting the samples at both at λ_ex_ = 257 and 276 nm.

### 3.7. Circular Dichroism

Far-UV CD spectra of DTPA(Gd)-PEG8-(FY)3 and DOTA(Gd)-PEG8-(FY)3 were carried out on a Jasco J-810 spectropolarimeter equipped with a NesLab RTE111 thermal controller unit using a 0.1 mm quartz cell at 25 °C. CD measurement were recorded from 280 to 190 nm at room temperature on samples at a concentration of 1.0 and 5.0 mg/mL. Other experimental settings were: Scan speed, 10 nm/min; sensitivity, 50 mdeg; time constant, 16 s; bandwidth, 1 nm. All the spectra were obtained by averaging three scans, after correction for the blank and for dilution. Ellipticities were reported as the mean residue ellipticity (MRE), which is the ellipticity per mole of peptide divided by the number of amino acid residues in the peptide.

### 3.8. Scanning Electron Microscopy (SEM)

A drop of hydrogel solution at 5 mg/mL concentration was deposited on glass cover-slips for microscopy and left to air-dry under ambient conditions. The samples were then coated with a 10 nm thick Au layer to facilitate conductance and imaged with a Scanning Electron Microscope (Zeiss, EVO50 XVP) equipped with Oxford Microanalysis system and LaB6 source. EHT was 15 kV and theoretical resolution 10 nm.

### 3.9. Fourier Transform Infrared Spectroscopy (FTIR)

Fourier Transform Infrared spectra of DTPA(Gd)-PEG8-(FY)3 and DOTA(Gd)-PEG8-(FY)3 in solution at the concentration of 5.0 mg/mL were collected on a Jasco FT/IR 4100 spectrometer (Easton, MD) in an attenuated total reflection (ATR) mode and using a Ge single-crystal at a resolution of 4 cm*^−^*^1^. Each sample was recorded with a total of 100 scans with a rate of 2 mm·s*^−^*^1^ against a KBr background. After collection in transmission mode, spectra were converted in emission.

### 3.10. Congo Red Spectroscopic Assay

UV-Vis measurements of Congo red (CR) alone or in presence of peptide conjugates (at a concentration of 1.5 mg/mL) were carried out on aforementioned spectrophotometer. Briefly 3.5 mg of CR powder were dissolved in 500 μL of 10 mM phosphate buffer at pH 7.4 and filtered through 0.2 μm syringe immediately before to use. Next, 5 μL of CR solution was diluted with the buffer at 25 μM final concentration and the UV-Vis spectrum was recorded in the range of wavelength between 400 and 700 nm at room temperature. Then 200 μL of the peptide solution (5.0 mg/mL) was added to CR solutions. The solutions containing peptide conjugates incubated with CR were left at room temperature for 30 min before recording the spectra.

### 3.11. Water Proton Relaxation Measurements

The longitudinal water proton relaxation rates were measured at 25 °C by using a Stelar Spinmaster (Stelar, Mede, Pavia, Italy) spectrometer operating at 0.5 T (21.5 MHz Proton Larmor Frequency), by mean of the standard inversion-recovery technique. The temperature was controlled with a Stelar VTC-91 air-flow heater equipped with a copper constantan thermocouple (uncertainty 0.1 °C). The proton 1/T_1_ NMRD profiles were measured at 25 °C on a fast field-cycling Stelar relaxometer over a continuum of magnetic field strengths from 0.00024 to 0.47 T (corresponding to 0.01–20 MHz proton Larmor frequencies). The relaxometer operates under computer control with an absolute uncertainty in 1/T_1_ of ±1%. Additional data points in the range 21.5–70 MHz were obtained on the Stelar Spinmaster spectrometer. The Gd-complexes concentrations in the solutions were determined according to a previously reported relaxometric method [43]. Data were fitted to the Solomon–Bloembergen–Morgan theory modified according to the Lipari-Szabo approach for the description of the rotational dynamics.

### 3.12. Cytotoxicity Studies

The cytotoxicity of DTPA(Gd)PEG8-(FY)3 and DOTA(Gd)PEG8-(FY)3 was investigated in the metastasizing TS/A mouse mammary adenocarcinoma cell line [44]. They were grown in RPMI (Roswell Park Memorial Institute)^1064^ medium supplemented with 10% heat-inactivated fetal bovine serum (FBS), 2 mM glutamine, 100 U/mL penicillin, and 100 µg/mL streptomycin. Cells were seeded in 75-cm^2^ flasks at density of ca. 2 × 10^4^ cells/cm^2^ in a humidified 5% CO_2_ incubator at 37 °C. At confluence, they were detached by adding 1 mL of Trypsin-EDTA solution (0.25% (w/v) Trypsin-0.53 mM EDTA). Cells were negative for mycoplasma as tested by using MycoAlert™ Mycoplasma Detection Kit. All cell media and supplements were purchased from Lonza Sales AG-EuroClone SpA, Milano (IT).

For cytotoxicity experiments, 8 × 10^4^ TS/A cells were seeded in 96-well plates the day before the incubation. The cells were incubated 4 h at 37 °C with the Gd-based probe at different concentration: 0.1, 0.25, 0.5, 1, 3, and 5 mg/mL (V = 0.1 mL). Cell viability was tested by using MTT assay (Sigma Aldrich). After incubation of TS/A cells, culture medium was removed and cells were cultured in presence of 0.1 mL of a 5 mg/mL MTT solution in fresh medium for 4 h, in a humidified 5% CO_2_ incubator at 37 °C. Then, the surnatant was removed and replaced with 0.1 mL of DMSO in order to dissolve the insoluble product into a colored solution. The absorbance of this colored solution (λ = 570 nm) was quantified by measuring using a BIORAD iMARK microplate-reader.

The experiments were carried out in triplicate and data reported as mean ±SD. As control, untreated TS/A cells were used. The percentage of viable cells was calculated according to the following equation:

$$Viable\;cells\;\left(\%\right)=\;\frac{Abs_{570nm}\;of\;tested\;cells}{Abs_{570nm}\;of\;untreated\;control\;cells}\;\times 100$$

Unpaired two-tails t-Student has been applied (* = *p*-value < 0.5, ** = *p*-value < 0.01).

## 4. Conclusions

Due to low cost, biocompatibility, tunable bioactivity, and chemical variety, nanostructures based on natural or unnatural peptides are promising molecules for the development of novel therapeutic or diagnostic agents. In this context, we previously reported several examples of fibrillary nanostructures as potential diagnostic agents for MRI applications. With the aim to enhance the contrast capability and reduce the cytotoxicity, we designed two novel peptide conjugates DTPA(Gd)-PEG8-(FY)3 or DOTA(Gd)-PEG8-(FY)3, in which the Gd-complex is positioned at the N-terminus of the polymer peptide PEG8-(FY)3. As previously reported for PEG8-(FY)3, both the peptide conjugates are able to generate soft hydrogels at a concentration of 0.5–1.0%wt. Below this concentration peptide begin to self-assemble. Moreover, the fine structural characterization, performed on the samples in solution with several spectroscopic techniques (CD, FTIR, UV-Vis and fluorescence), highlighted their tendency to self-assemble into β-sheet structures with an antiparallel orientation of the β-strands. On the other hand, SEM microphotos revealed a difference in the morphology of the aggregates. These findings suggest that the derivatization of the N-terminus of the peptide-polymer with Gd-complexes DOTA(Gd) or DTPA(Gd) does not prevent the supramolecular organization of the peptide building block. This result could be expected taking into account the presence of the PEG spacer between the peptide moiety and chelating agent. In other words, we can conclude that the PEG prevents the aggregate from unfavorable steric and electrostatic interactions occurring between chelating agents. If the PEG is able to safeguard the peptide structural organization, it is also responsible for the very low τ_Rl_ (75 and 46 ps for DTPA and DOTA, respectively) values that in turn affect the measured relaxivity values (~12 mM*^−^*^1^ s*^−^*^1^ at 20 MHz). However, it is worth to note that this relaxivity is 2–3 fold higher than that of the corresponding low molecular weight CAs. The low cytotoxicity of both the peptide conjugates on the metastasizing TS/A mouse mammary adenocarcinoma cell line selected for this study is favorable for potential application in medicine as supramolecular diagnostic agents for MRI.

## Figures and Tables

**Table 1:** Main relaxometric parameters derived from fitting of NMRD profiles reported in Figure 4 and Figure 5a.

System	r_1_ (mM^−1^s^−1^) at 21.5 MHz, 25 °C	Δ^2^(s^−2^) [a]	*τ*_V_ (ps) [b]	*τ*_R_^l^ (ps) [c]	*τ*_R_^g^ (ps) [c]	S^2^ [d]
DTPA(Gd)-PEG8-(FY)3	10.5	8.75 × 10^18^	51	76	1718	0.44
0.5 mg/mL, pH = 7						
DOTA(Gd)-PEG8-(FY)3	8.6	8.29 × 10^18^	45	45	2060	0.36
0.5 mg/mL, pH = 7						
DTPA(Gd)-PEG8-(FY)3	12.0	1.32 × 10^19^	46	330	3378	0.32
5 mg/mL, pH = 7						
DOTA(Gd)-PEG8-(FY)3	12.1	9.60 × 10^18^	46	224	2690	0.40
10 mg/mL, pH = 7						
DTPA(Gd)-PEG8-(FY)3	13.1	7.97 × 10^18^	57	293	2270	0.44
0.5 mg/mL, pH = 3						
DTPA(Gd)-PEG8-(FY)3	13.5	8.15 × 10^18^	55	400	2480	0.41
0.5 mg/mL, pH = 11						

On carrying out the fitting procedure, some parameters were fixed to reasonable values: r_Gd-H_ (distance between Gd and protons of the inner sphere water molecule) = 3.1 Å; a (distance of minimum approach of solvent water molecules to Gd^3+^ ion) = 3.8 Å; D (solvent diffusion coefficient) = 2.2∙10^−5^ cm^2^ s^−1^. (a) Squared mean transient zero-field splitting (ZFS) energy. (b) Correlation time for the collision-related modulation of the ZFS Hamiltonian. (c) Reorientational correlation time. (d) Order parameter.

## Supplementary Materials

The following are available online at https://www.mdpi.com/1424-8247/13/2/19/s1, Figure S1: Chromatograms and ESI spectra of DOTA-PEG8-(FY)3 and DTPA-PEG8-(FY)3. Figure S2: Fluorescence emission spectra of DTPA(Gd)-PEG8-(FY)3 excited at λex = 276 nm in 0.1-2.5 mg/mL concentration range. Figure S3: FTIR spectra of DTPA-PEG8-(FY)3 as free base (in black) and as Gd-complex (in red).
