# Supplementary material for: Peptide-Based Soft Hydrogels Modified with Gadolinium Complexes as MRI Contrast Agents
Source: Pharmaceuticals (Basel). 2020 Jan 21;13(2):19. doi: 10.3390/ph13020019 (PMC7168922; doi:10.3390/ph13020019)
Supplement: Supplementary file 1 [file pharmaceuticals-13-00019-s001.pdf]

DOTA-PEG8-(FY)3

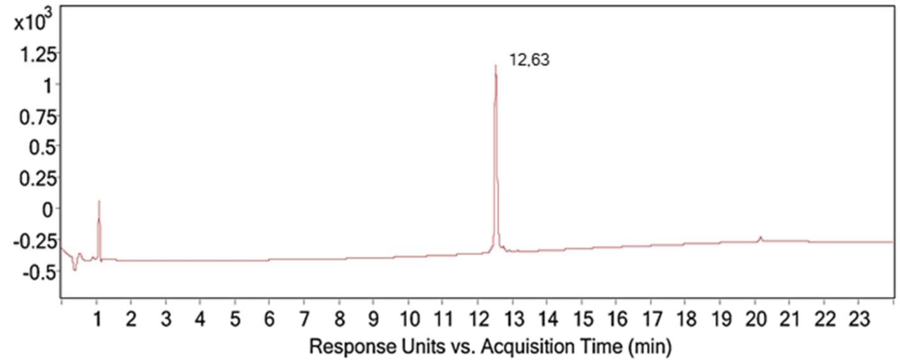

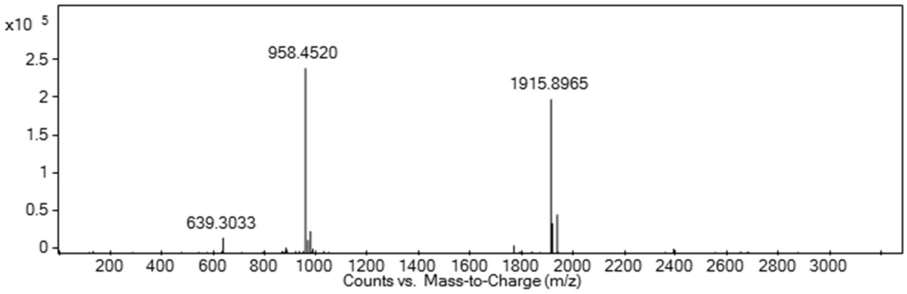

DTPA-PEG8-(FY)3

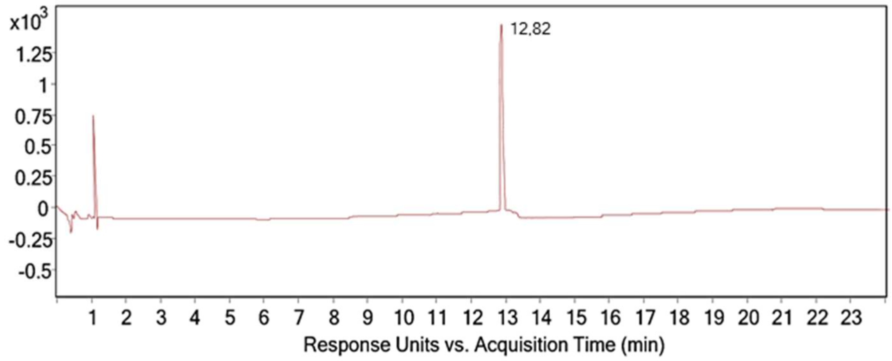

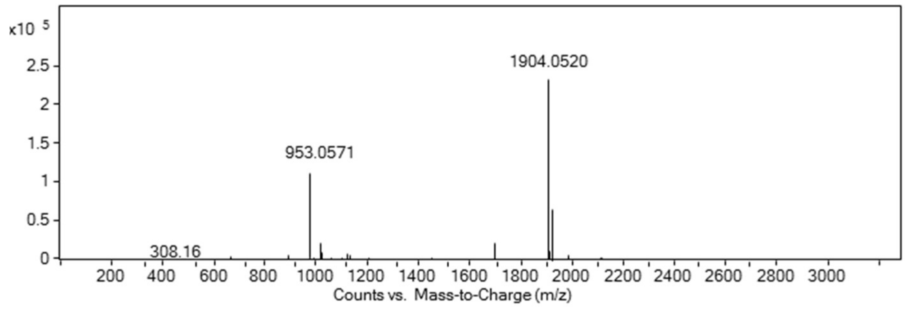

Figure S1: Chromatograms and ESI spectra of DOTA-PEG8-(FY)3 and DTPA-PEG8-(FY)3.

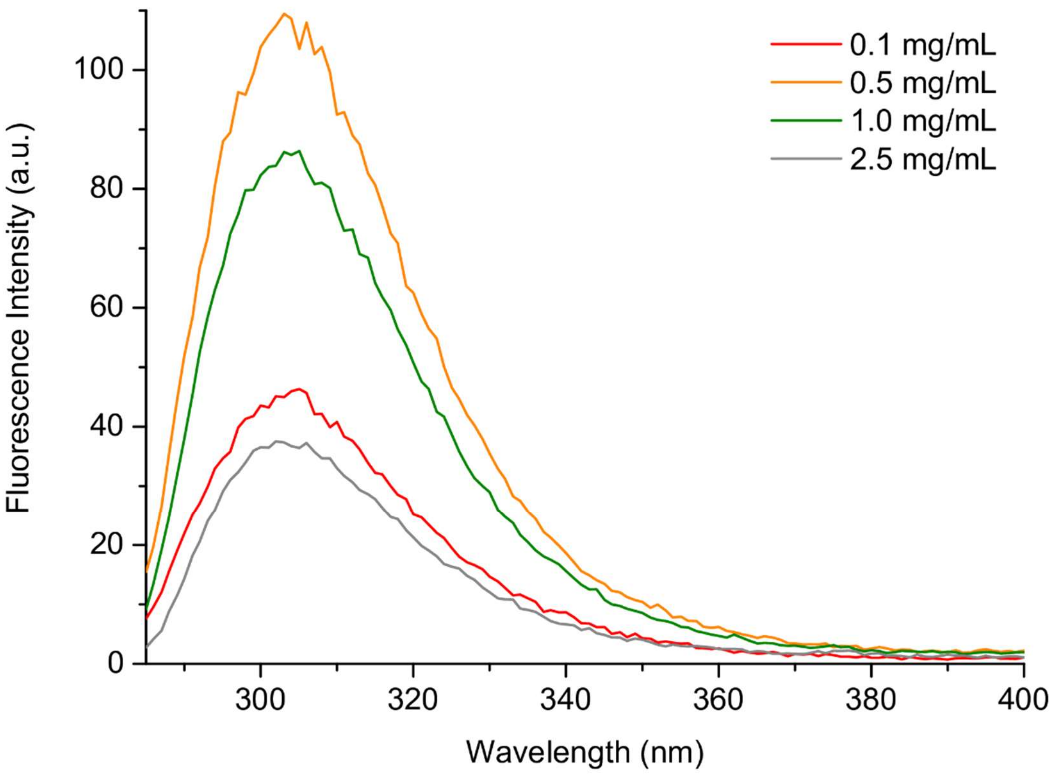

Figure S2: Fluorescence emission spectra of DTPA(Gd)-PEG8-(FY)3 excited at λex = 276 nm in 0.1-2.5 mg/mL concentration range.

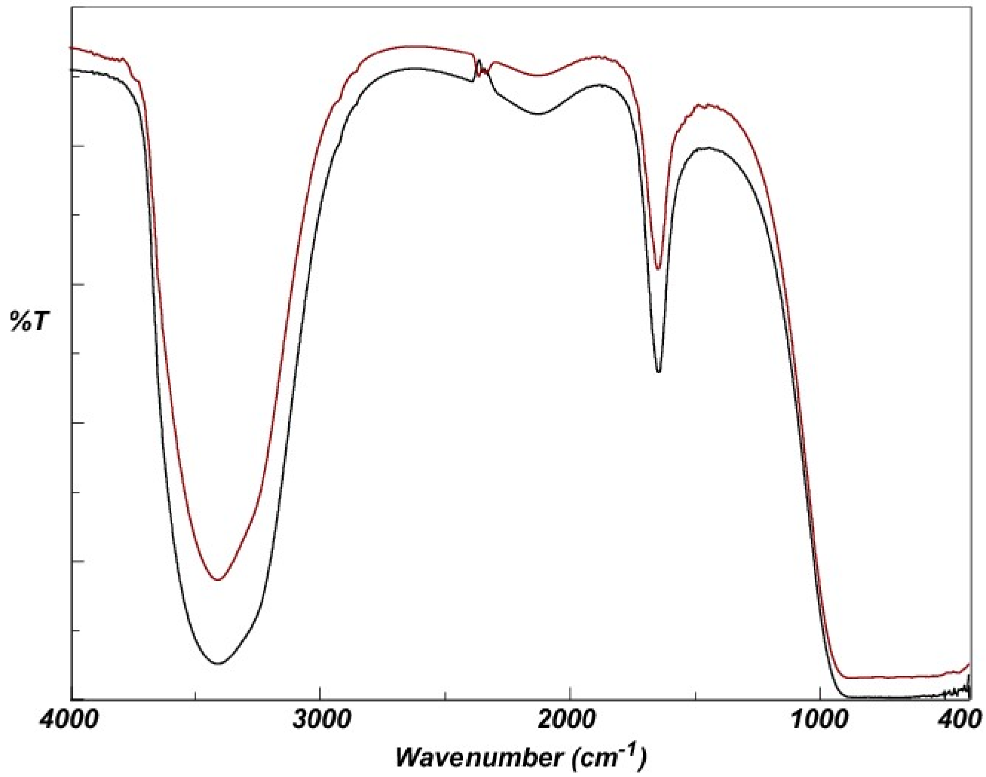

Figure S3: FTIR spectra of DTPA-PEG8-(FY)3 as free base (in black) and as Gd-complex (in red).
